# Disaggregation of Breastfeeding Initiation Rates by Race and Ethnicity — United States, 2020–2021

**DOI:** 10.5888/pcd20.230199

**Published:** 2023-12-14

**Authors:** Kristin J. Marks, Jasmine Y. Nakayama, Katelyn V. Chiang, Mary Ellen Grap, Erica H. Anstey, Ellen O. Boundy, Heather C. Hamner, Ruowei Li

**Affiliations:** 1 Epidemic Intelligence Service, Centers for Disease Control and Prevention, Atlanta, Georgia; 2 Division of Nutrition, Physical Activity, and Obesity, Centers for Disease Control and Prevention, Atlanta, Georgia; 3 US Public Health Service, Rockville, Maryland; 4 Nutrition and Health Sciences Program, Laney Graduate School, Emory University, Atlanta, Georgia; 5 Oak Ridge Institute for Science and Education, Oak Ridge, Tennessee

## Abstract

**Introduction:**

Although breastfeeding is the ideal source of nutrition for most infants, racial and ethnic disparities exist in its initiation. Surveillance rates based on aggregated data can challenge the understanding and monitoring of effective, culturally appropriate interventions among racial and ethnic subgroups. Aggregated data have historically estimated breastfeeding rates among a few large racial and ethnic groups. We examined differences in breastfeeding initiation rates by disaggregation of data to finer subgroups of race and ethnicity.

**Methods:**

We analyzed births from January 1, 2020, through December 31, 2021, in 48 states and the District of Columbia by using National Vital Statistics System birth certificate data. Data indicate whether an infant received any breast milk during birth hospitalization and include self-reported maternal race and ethnicity. Cross-tabulations of race and ethnicity by breastfeeding initiation were calculated and compared across aggregated and disaggregated categories.

**Results:**

The overall prevalence of breastfeeding initiation was 84.0%, ranging from 74.5% (mothers identifying as Black) to 94.0% (mothers identifying as Japanese). The aggregated prevalence of breastfeeding initiation among mothers identifying as Hispanic was 86.8%; disaggregated estimates by Hispanic origin ranged from 82.2% (Puerto Rican) to 90.9% (Cuban).

**Conclusion:**

Substantial variation in the prevalence of breastfeeding initiation across disaggregated racial or ethnic categories exists. Disaggregation of racial and ethnic data unmasked differences that could reflect variations in cultural practices or systemic barriers to breastfeeding. Understanding why these differences exist could guide public health practitioners’ efforts to improve and tailor breastfeeding support.

**Summary**

**What is already known on this topic?**

Racial and ethnic disparities exist in breastfeeding initiation. Aggregated data can mask health disparities.

**What is added by this report?**

The aggregated prevalence of breastfeeding initiation was highest among mothers identifying as Asian (90.1%); however, disaggregated estimates among Asian subgroups varied widely (84.2%–94.0%). A wide range was also seen among mothers identifying as Hispanic.

**What are the implications for public health practice?**

Data disaggregation paints a more detailed picture of health. Disaggregation can ensure that populations that have been historically masked in public health surveillance and research are more visible, allowing approaches to health services that address specific needs and create solutions to eliminate health disparities in outcomes such as breastfeeding initiation.

## Introduction

Breastfeeding is the optimal source of nutrition for most infants (1) and can reduce the risk for several maternal and infant health conditions (2,3). Although US breastfeeding rates have increased over time (4), racial and ethnic disparities persist (5,6). Recent data indicate that breastfeeding initiation rates are higher (≥80%) among infants of mothers who identify as Asian, Hispanic, or White and lower (<80%) among those who identify as American Indian or Alaskan Native (AI/AN), Black, or Native Hawaiian or Other Pacific Islander (NHOPI) (5,7).

Population-level health indicators are often described relative to broad racial and ethnic categories. The aggregation of heterogeneous data can obscure within-group differences (8), limiting the understanding of health disparities among diverse subpopulations, including Hispanic, Asian, and Native Hawaiian or Other Pacific Islander populations (9–14). However, guidance on data disaggregation has varied and changed over time (15–17). In 1997, the US Office of Management and Budget (OMB) disaggregated the “Asian or Pacific Islander” category into 2 separate categories, Asian and Native Hawaiian or Other Pacific Islander, creating 5 minimum categories for data on race (AI/AN, Asian, Black, NHOPI, and White) (15) which are often used. Given the diversity within broad racial and ethnic groups, health research is moving toward data disaggregation (8).

The aggregated reporting of race and ethnicity can fail to capture diversity in languages, cultural practices, and in social, economic, and environmental experiences. Historically, barriers to data disaggregation include small numbers among subpopulations and lack of space on data collection instruments (18). Without disaggregated data, health disparities might be masked, and research, programs, and policies might be misdirected. To investigate the extent to which broad categories of race and ethnicity mask variation across subpopulations, we examined differences in breastfeeding initiation rates within racial and ethnic groups by disaggregation to finer subgroups by using birth certificate data from 48 states and the District of Columbia.

## Methods

### Data source

**Figure 1:**
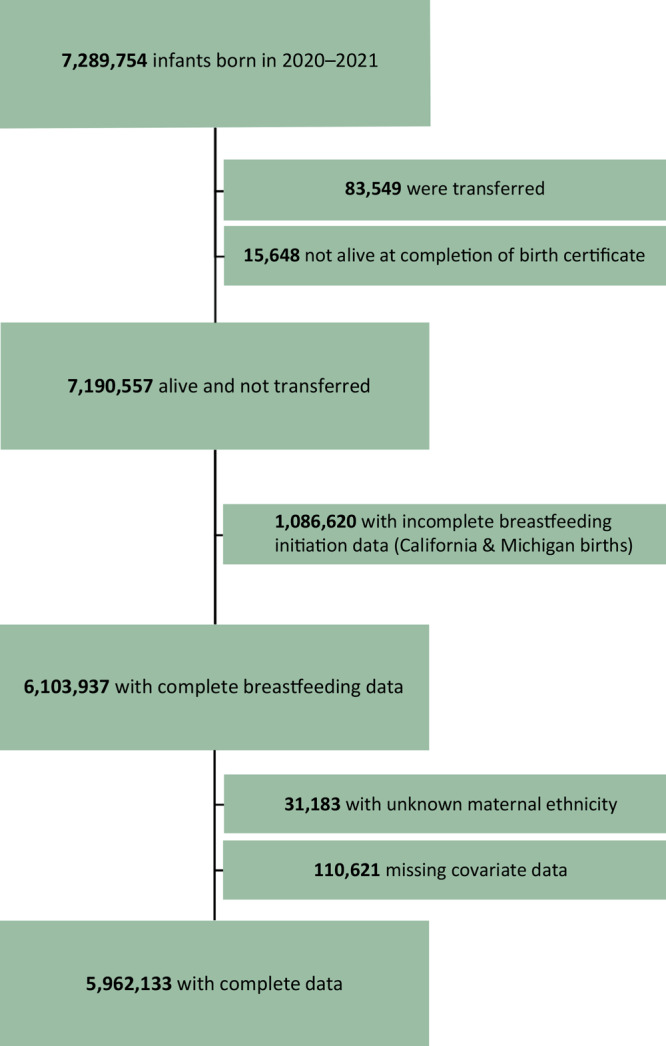
Flow of participants in an analysis of disaggregated breastfeeding initiation rates by race and ethnicity, United States, 2020–2021, using National Vital Statistics System birth certificate data from 2020–2021 US births. Source: National Vital Statistics System (19).

The National Vital Statistics System led by the National Center for Health Statistics (NCHS) is a census of all live births in the United States and is derived from the Standard Certificate for Live Birth (19). By 2016, all 50 states and the District of Columbia adopted the 2003 revision of the birth certificate, which includes a question regarding breastfeeding initiation. Birth certificate data are collected from information provided by the mother and medical records during the period from delivery to discharge from the birth facility or completion of the birth certificate for home births (20). In the present analysis, all infants born from January 1, 2020, through December 31, 2021, in 48 of the 50 states (California was excluded because breastfeeding data are not reported to NCHS and Michigan, because data are collected inconsistently) and the District of Columbia are included. Infants were excluded from the analysis if they were transferred to another facility within 24 hours of delivery, died before completion of the birth certificate, or were missing breastfeeding initiation or covariate data (Figure 1) (21). Births in these 48 states and the District of Columbia represent 85% of US live births. Our final analytic sample included 5,962,133 infants born in 2020 and 2021. We used the publicly available downloadable file to access the data (21).

### Exposures

Birth certificate data include self-reported maternal race and ethnicity (22). For the birth certificate, mothers can select 1 or more races from 15 categories, with the option to write in greater detail for a few categories (eg, name of the enrolled or principal tribe for those selecting American Indian or Alaskan Native). NCHS aggregates these 15 categories into 6 broader categories of maternal race: American Indian or Alaskan Native, Asian (Asian Indian, Chinese, Filipino, Japanese, Korean, Vietnamese, and other Asian), Black or African American (hereinafter Black), Native Hawaiian or Other Pacific Islander (Native Hawaiian, Guamanian or Chamorro, Samoan, and other Pacific Islander), White, and more than 1 race. A 31-level variable with greater detail on multiple race identities is also publicly available, in addition to the 6- and 15-level variables. Of note, more detailed maternal race data are available via restricted-use access.

Similarly, mothers are asked to self-report their Hispanic origin as part of the birth certificate. Public-use data files include 8 categories of maternal ethnicity: non-Hispanic, Mexican, Puerto Rican, Cuban, Central or South American, Dominican, other and unknown Hispanic origin, and origin unknown or not stated. NCHS classifies mothers as Hispanic if they identify as Mexican, Puerto Rican, Cuban, Central or South American, Dominican, and Other and Unknown Hispanic origin. We created an aggregated maternal ethnicity variable with 2 categories of Hispanic and non-Hispanic to demonstrate the effect of aggregation.

### Outcome

Breastfeeding initiation was collected on the birth certificate by answering the question “Is the infant being breastfed at discharge?” with a yes or no response option. NCHS provides detailed guidance to assist in completion of the facility worksheet for the birth certificate. Instructions specify that breastfeeding should be determined from medical records and from indication of receipt of any breast milk or colostrum during the period from birth to discharge from the hospital (including donor milk) (20). Information regarding the duration or exclusivity of breastfeeding or supplementation with formula is not available through birth certificate data. Those who had a “yes” response to the question were considered to have initiated breastfeeding.

### Statistical analyses

We reported univariate descriptive statistics for the distribution of 2020 and 2021 US births by using the previously described 15 categories of maternal race and 8 categories of maternal ethnicity. We also reported the prevalence of breastfeeding initiation by these categories with 95% CIs. We compared the prevalence of breastfeeding initiation among mothers identifying as Asian, Native Hawaiian or Other Pacific Islander, or Hispanic to their more detailed subgroups to determine whether subgroup prevalence estimates differed from their corresponding aggregated estimates. To compute the range, we calculated the prevalence of breastfeeding initiation among subgroups of mothers identifying as Asian, Native Hawaiian or Other Pacific Islander, or Hispanic by using the highest and lowest prevalence estimate of breastfeeding initiation in the disaggregated subgroups.

We calculated the adjusted prevalence and prevalence ratios of breastfeeding initiation of Asian, Native Hawaiian or Other Pacific Islander, and Hispanic subgroups to explore whether within-group differences could be explained by differences in other maternal and infant characteristics across the subgroups. We calculated these adjusted prevalence estimates and prevalence ratios by using predicted marginal values from logistic regression models that controlled for maternal and infant characteristics postulated a priori to be associated with both maternal race or ethnicity and breastfeeding initiation (23). In prevalence ratios, the racial or ethnic group with the lowest prevalence of breastfeeding initiation was used as the reference group.

Models were adjusted for maternal age (<25 y, 25–34 y, ≥35 y), maternal education (≤high school graduate, some college, ≥Bachelor’s degree), maternal nativity (born in the US, born outside the US), marital status (married, unmarried), parity (nulliparous, parous), participation in Special Supplemental Nutrition Program for Women, Infants, and Children (WIC) during pregnancy (yes, no), delivery method (vaginal, Cesarean), and infant sex (male, female). We then compared the range of adjusted prevalence estimates among Asian, Native Hawaiian or Other Pacific Islander, and Hispanic subgroups to the range of unadjusted prevalence estimates.

In supplemental analyses, we described breastfeeding initiation rates by using detailed data on the multiracial identities of mothers. Data were suppressed for any racial or ethnic group with a denominator less than 50.

Analyses were conducted by using SAS version 9.4 (SAS Institute Inc) and SAS-callable SUDAAN software (RTI). The Centers for Disease Control and Prevention determined that this study was not subject to institutional review board review because only publicly available, deidentified secondary data were analyzed.

## Results

Among 5,962,133 infants in our study who were born in the United States in 2020 and 2021, most were born to mothers who self-identified their race as White (73.7%), followed by mothers who identified as Black (17.1%), and mothers who identified as Asian (5.2%) (Table 1). Among mothers who identified as Asian, those who identified as Asian Indian accounted for more than one-third of Asian births (1.8% of all births). We also examined the distribution of births by Hispanic ethnicity and found that 21.8% of mothers self-identified their ethnicity as Hispanic, with half of those mothers identifying as Mexican (10.9% of all births) and another one-fifth identifying as Central or South American (4.6% of all births). Most mothers who identified as Hispanic identified their race as White (88.7%) or Black (7.3%).

**Table 1:** Distribution of Births By Maternal Race and Maternal Ethnicity, With Detailed Asian, Native Hawaiian or Other Pacific Islander, and Hispanic Subgroups^a^ — National Vital Statistics System, 48 States^b^ and District of Columbia, 2020–2021

Race	Total^c^	Ethnicity	
		Non-Hispanic	Hispanic^c^
**Total**	5,962,133 (100.0)	4,661,133 (78.2)	1,301,000 (21.8)
**Maternal race^d^**			
**White**	4,392,277 (73.7)	3,238,217 (69.5)	1,154,060 (88.7)
**Black or African American**	1,020,169 (17.1)	925,105 (19.8)	95,064 (7.3)
**Asian**	307,956 (5.2)	297,765 (6.4)	10,191 (0.8)
Asian Indian	105,072 (1.8)	103,520 (2.2)	1,552 (0.1)
Other Asian	70,175 (1.2)	67,062 (1.4)	3,113 (0.2)
Chinese	49,174 (0.8)	48,149 (1.0)	1,025 (0.08)
Filipino	34,499 (0.6)	31,797 (0.7)	2,702 (0.2)
Vietnamese	25,225 (0.4)	24,514 (0.5)	711 (0.05)
Korean	16,693 (0.3)	16,088 (0.3)	605 (0.05)
Japanese	7,118 (0.1)	6,635 (0.1)	483 (0.04)
**More than one race**	161,554 (2.7)	138,997 (3.0)	22,557 (1.7)
**American Indian and Alaskan Native**	60,511 (1.0)	46,700 (1.0)	13,811 (1.1)
**Native Hawaiian or Other Pacific Islander **	19,666 (0.3)	14,349 (0.3)	5,317 (0.4)
Other Pacific Islander	12,069 (0.2)	8,658 (0.2)	3,411 (0.3)
Samoan	3,170 (0.05)	2,911 (0.06)	259 (0.02)
Guamanian or Chamorro	2,540 (0.04)	1,403 (0.03)	1,137 (0.09)
Hawaiian	1,887 (0.03)	1,377 (0.03)	510 (0.04)
**Maternal ethnicity^d^**			
**Non-Hispanic**	4,661,133 (78.2)	Not applicable	
**Hispanic**	1,301,000 (21.8)		
Mexican	650,486 (10.9)		
Central or South American	274,510 (4.6)		
Other and Unknown Hispanic Origin	139,969 (2.4)		
Puerto Rican	129,259 (2.2)		
Dominican	62,166 (1.0)		
Cuban	44,610 (0.8)		

a Excludes infants transferred to another facility within 24 hours of delivery and those who died before completion of the birth certificate.

b Includes all states except California and Michigan.

c Values are number (percentage).

d Categories are listed by number of births, starting with the highest number of births.

**Figure 2:**
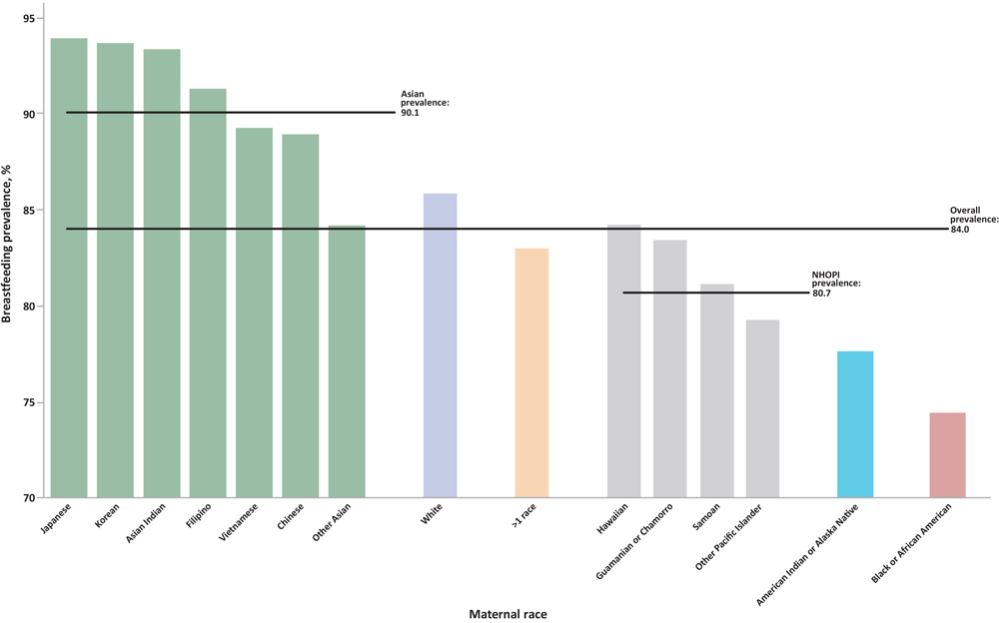
Breastfeeding initiation by maternal racial groups, with disaggregation of Asian and Native Hawaiian or Other Pacific Islander subgroups, for 48 states and the District of Columbia, 2020–2021. Two states were excluded: California, because breastfeeding data are not reported to NCHS, and Michigan, because data are collected inconsistently. Source: National Vital Statistics System (19).

The overall prevalence of breastfeeding initiation was 84.0%, although this varied by almost 10 percentage points in each direction across the 15 categories of maternal race (Figure 2). The lowest prevalence of breastfeeding initiation was among mothers who identified as Black (74.5%). Two other racial subgroups also had prevalence estimates of less than 80% initiation, American Indian or Alaskan Native (77.7%) and Other Pacific Islanders (79.4%). The highest prevalence of breastfeeding initiation was among mothers who identified as Japanese (94.0%), followed by mothers who identified as Korean (93.7%), Asian Indian (93.4%), and Filipino (91.3%); all 4 of these disaggregated subgroups are part of the aggregated Asian racial group.

**Table:** 

Race	Subgroup estimate	Group average	Overall prevalence
Japanese	94.0	90.1	84.1
Korean	93.7	90.1	84.1
Asian Indian	93.4	90.1	84.1
Filipino	91.3	90.1	84.1
Vietnamese	89.3	90.1	84.1
Chinese	89.0	90.1	84.1
Other Asian	84.2	90.1	84.1
White	Not applicable	85.9	84.1
>1 race	Not applicable	83.1	84.1
Hawaiian	84.3	80.7	84.1
Guamanian or Chamorro	83.5	80.7	84.1
Samoan	81.2	80.7	84.1
Other Pacific Islander	79.4	80.7	84.1
American Indian or Alaskan Native	Not applicable	77.7	84.1
Black or African American	Not applicable	74.5	84.1

**Figure 3:**
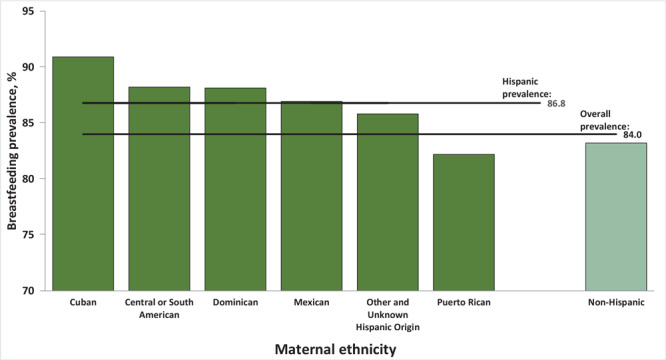
Breastfeeding initiation by maternal ethnicity groups, with disaggregation of Hispanic ethnicity subgroups, for 48 states and the District of Columbia, 2020-2021. Two states were excluded: California, because breastfeeding data are not reported to NCHS and Michigan, because data are collected inconsistently. Source: National Vital Statistics System (19).

Of mothers who identified as Hispanic 86.8% initiated breastfeeding (Figure 3). Among those mothers, those who identified as Cuban were most likely to initiate breastfeeding (90.9%), whereas mothers who identified as Puerto Rican were the least likely (82.2%).

**Table:** 

Hispanic ethnicity	Subgroup	Group Average	Overall average
Cuban	90.9	86.8	84.0
Central or South American	88.2	86.8	84.0
Dominican	88.1	86.8	84.0
Mexican	86.9	86.8	84.0
Other and Unknown Hispanic Origin	85.8	86.8	84.0
Puerto Rican	82.2	86.8	84.0
Non-Hispanic	Not applicable	83.2	84.0

Among mothers who identified as Asian, aggregated prevalence estimates of breastfeeding initiation were 90.1%; however, disaggregated prevalence estimates varied from 94.0% (Japanese) to 84.2% (Other Asian), a range of 9.8 percentage points and a prevalence ratio (PR) for Japanese mothers of 1.12 (95% CI, 1.11–1.12) (Table 2). When adjusting for maternal and infant characteristics, the range narrowed to 5.0 percentage points and an adjusted prevalence ratio (aPR) of 1.06 (95% CI, 1.05–1.07). Similarly, aggregated prevalence estimates of breastfeeding initiation among mothers identifying as Hispanic were 86.8%; however, disaggregated prevalence estimates had a range of 8.7 percentage points (PR, 1.11; 95% CI, 1.10–1.11) but narrowed to 5.7 percentage points (aPR, 1.07; 95% CI, 1.06–1.07) following adjustment for maternal and infant characteristics. Disaggregated estimates within the Native Hawaiian or Other Pacific Islander group had a spread of 4.9 percentage points before adjustment (PR, 1.06; 95% CI, 1.04–1.08)) and 3.3 percentage points after adjustment (aPR, 1.04; 95% CI, 1.02–1.07).

**Table 2:** Unadjusted and Adjusted^a^ Prevalence of Breastfeeding Initiation by Disaggregated Maternal Race and Maternal Ethnicity Subgroups^b^ — National Vital Statistics System, 48 States^c^ and the District of Columbia, 2020–2021

Variable	Unadjusted prevalence of breastfeeding initiation, % (95% CI)	Unadjusted prevalence ratio of breastfeeding initiation, PR (95% CI)	Adjusted prevalence of breastfeeding initiation, % (95% CI)^d^	Adjusted prevalence ratio of breastfeeding initiation, PR (95% CI)
**Maternal race^e^**				
**Asian**				
Japanese	94.0 (93.4–94.6)	1.12 (1.11–1.12)	93.0 (92.4–93.6)	1.06 (1.05–1.07)
Korean	93.7 (93.4–94.1)	1.11 (1.11–1.12)	91.8 (91.3–92.2)	1.04 (1.04–1.05)
Asian Indian	93.4 (93.3–93.6)	1.11 (1.11–1.11)	91.8 (91.6–92.0)	1.04 (1.04–1.05)
Filipino	91.3 (91.0–91.6)	1.08 (1.08–1.09)	92.0 (91.7–92.2)	1.05 (1.04–1.05)
Vietnamese	89.3 (89.0–89.7)	1.06 (1.06–1.07)	90.9 (90.5–91.1)	1.03 (1.03–1.04)
Chinese	89.0 (88.7–89.3)	1.06 (1.05–1.06)	87.9 (87.6–88.2)	1.00 (1.00–1.00)
Other Asian	84.2 (84.0–84.5)	Reference	88.0 (87.7–88.2)	Reference
Range^f^	9.8	Not applicable	5.0	Not applicable
**Native Hawaiian or Other Pacific Islander**				
Hawaiian	84.3 (82.6–85.9)	1.06 (1.04–1.08)	82.3 (80.4–84.1)	1.03 (1.00–1.06)
Guamanian or Chamorro	83.5 (82.0–84.9)	1.05 (1.03–1.07)	83.0 (81.5–84.4)	1.04 (1.02–1.07)
Samoan	81.2 (79.8–82.6)	1.02 (1.00–1.04)	79.7 (78.2–81.1)	Reference
Other Pacific Islander	79.4 (78.6–80.1)	Reference	80.2 (79.5–80.9)	1.01 (0.99–1.03)
Range^f^	4.9	Not applicable	3.3	Not applicable
**Maternal ethnicity^e^**				
**Hispanic**				
Cuban	90.9 (90.6–91.2)	1.11 (1.10–1.11)	88.8 (88.5–89.1)	1.07 (1.06–1.07)
Central or South American	88.2 (88.1–88.3)	1.07 (1.07–1.08)	86.7 (86.6–86.9)	1.04 (1.04–1.05)
Dominican	88.1 (87.9–88.4)	1.07 (1.07–1.08)	86.0 (85.7–86.3)	1.04 (1.03–1.04)
Mexican	86.9 (86.9–87.0)	1.06 (1.05–1.06)	87.6 (87.5–87.6)	1.05 (1.05–1.06)
Other and Unknown Origin	85.8 (85.6–86.0)	1.04 (1.04–1.05)	86.6 (86.5–86.8)	1.04 (1.04–1.05)
Puerto Rican	82.2 (82.0–82.4)	Reference	83.1 (82.9–83.3)	Reference
Range^f^	8.7	Not applicable	5.7	Not applicable

Abbreviation: PR, prevalence ratio.

a Models are adjusted for maternal age, maternal education, maternal nativity, marital status, parity, participation in Special Supplemental Nutrition Program for Women, Infants, and Children during pregnancy, delivery method, and infant sex.

b Excludes infants transferred to another facility within 24 hours of delivery and those who died before completion of the birth certificate.

c Includes all states except California and Michigan.

d We were unable to calculate adjusted prevalence estimates for the aggregated racial or ethnic groups because the method used requires the specification of a reference group. We used the predicted marginal approach to calculate adjusted prevalence estimates among mothers identifying as Asian, Native Hawaiian or Other Pacific Islander, and Hispanic, meaning we subset to these separate groups and adjusted to their distributions.

e Racial groups not listed in this table include White, Black, American Indian or Alaskan Native, and multiracial. The ethnic group not listed in the table is non-Hispanic.

f To compute the range, we calculated the prevalence of breastfeeding initiation among subgroups of mothers identifying as Asian, Native Hawaiian or Other Pacific Islander, or Hispanic by using the highest and lowest prevalence estimate of breastfeeding initiation in the disaggregated subgroups. Values are percentage points.

We examined the prevalence of breastfeeding initiation by using detailed information about mothers who identified as multiracial (Table 3). Breastfeeding initiation rates among mothers who identified as American Indian or Alaskan Native, Asian, and Native Hawaiian or Other Pacific Islander (94.2%), Asian and White (92.3%), and American Indian or Alaskan Native, Asian, and White (90.4%) exceeded that of the single aggregated racial group with the highest rate (Asian, 90.1%). Three multiracial subgroups, mothers who identified as Black and American Indian or Alaskan Native (79.7%), Black and White (77.9%), and Black, American Indian or Alaskan Native, and Native Hawaiian or Other Pacific Islander (75.0%), had breastfeeding initiation rates below 80%, though these rates were still above those of the single aggregated racial group (Black, 74.5%) with the lowest initiation rate.

**Table 3:** Distribution of Births by Maternal Race With Detailed Multiracial Subgroups^a^, National Vital Statistics System, 48 States^b^ and the District of Columbia, 2020–2021

Maternal race	Total	Initiated breastfeeding
	N (%)	n (%)
**All races**	5,962,133 (100.0)	5,008,132(84.0)
**American Indian or Alaskan Native (only)**	60,511 (1.0)	46,988 (77.7)
**Asian (only)**	307,956 (5.2)	277,383 (90.1)
**Black (only)**	1,020,169 (17.1)	760,467 (74.5)
**Native Hawaiian or Other Pacific Islander (only)**	19,666 (0.3)	15,861 (80.7)
**White (only)**	4,392,277 (73.7)	3,773,218 (85.9)
**More than one race**	161,554 (2.7)	134,215 (83.1)
American Indian/Alaskan Native, Asian, and Native Hawaiian or Other Pacific Islander	52 (<0.1)	49 **(**94.2)
Asian and White	30,149 (0.5)	27,841 (92.3)
American Indian or Alaskan Native, Asian, and White	715 (<0.1)	646 (90.4)
Asian, Native Hawaiian or Other Pacific Islander, and White	6,205 (0.1)	5,534 (89.2)
American Indian or Alaskan Native, Native Hawaiian or Other Pacific Islander, and White	167 (<0.1)	147 (88.0)
Black, American Indian or Alaskan Native, Native Hawaiian or Other Pacific Islander, and White	58 (<0.1)	51 (87.9)
Native Hawaiian or Other Pacific Islander and White	3,956 (0.1)	3,470 (87.7)
Asian and Native Hawaiian or Other Pacific Islander	3,378 (0.1)	2,960 (87.6)
Black and Asian	3,940 (0.1)	3,451 (87.6)
Black, American Indian or Alaskan Native, Asian, Native Hawaiian or Other Pacific Islander, and White	56 (<0.1)	49 (87.5)
American Indian or Alaskan Native, Asian, Native Hawaiian or Other Pacific Islander, and White	343 (<0.1)	298 (86.9)
Black, Asian, and White	1,625 (<0.1)	1,403 (86.3)
American Indian or Alaskan Native and Asian	630 (<0.1)	542 (86.0)
Black, American Indian or Alaskan Native, Asian, and White	252 (<0.1)	216 (85.7)
Black and Native Hawaiian or Other Pacific Islander	959 (<0.1)	813 (84.8)
American Indian or Alaskan Native and Native Hawaiian or Other Pacific Islander	200 (<0.1)	168 (84.0)
Black, Asian, Native Hawaiian or Other Pacific Islander, and White	175 (<0.1)	147 (84.0)
American Indian or Alaskan Native and White	25,236 (0.4)	21,131 (83.7)
Black, Native Hawaiian or Other Pacific Islander, and White	291 (<0.1)	240 (82.5)
Black, American Indian or Alaskan Native, and White	4,695 (0.1)	3,853 (82.1)
Black, Asian, and Native Hawaiian or Other Pacific Islander	193 (<0.1)	156 (80.8)
Black, American Indian or Alaskan Native, and Asian	170 (<0.1)	136 (80.0)
Black and American Indian or Alaskan Native	4,992 (0.1)	3,980 (79.7)
Black and White	73,036 (1.2)	56,875 (77.9)
Black, American Indian or Alaskan Native, and Native Hawaiian or Other Pacific Islander	64 (<0.1)	48 (75.0)
Black, American Indian or Alaskan Native, Asian, and Native Hawaiian or Other Pacific Islander	—c	—c

a Excludes infants transferred to another facility within 24 hours of delivery and those who died before completion of the birth certificate.

b Includes all states except California and Michigan.

c Data were suppressed for any racial or ethnic group with a denominator less than 50.

## Discussion

To investigate the potential impact of aggregating race and ethnicity data, we examined a sample of almost 6 million US births from 2021 and 2022, reported breastfeeding initiation rates by subgroups of Asian and Native Hawaiian or Other Pacific Islander race and Hispanic ethnicity, and compared these disaggregated rates with aggregated rates. We found considerable variation in the prevalence of breastfeeding initiation across these disaggregated racial and ethnic categories, suggesting that aggregation of race and ethnicity data could lead to over- or underestimation of breastfeeding initiation, if used as a proxy for some subgroups. Disaggregation of data unmasked differences that may suggest variations in cultural practices or help elucidate systemic barriers or facilitators to breastfeeding. These disaggregated rates can be used to celebrate strengths and to identify groups that may benefit from focused interventions. The latter could result in increased breastfeeding overall and reduced racial or ethnic disparities in breastfeeding.

Breastfeeding initiation is one example of the importance of data disaggregation. In our study, we observed that the aggregation of racial and ethnic groups obscured subgroups, which may benefit from additional breastfeeding education and support. In the aggregated Asian and Hispanic groups, one subgroup in each (Other Asian and Puerto Rican, respectively) had notably lower breastfeeding initiation rates than other subgroups and the aggregated breastfeeding initiation rate. Of note, aggregated estimates are weighted averages influenced by the sample size of each subgroup. For example, we saw that the aggregated prevalence of breastfeeding initiation among mothers who identified as Hispanic was 86.8%. This aggregated estimate is driven by the prevalence among mothers who identified as Mexican (86.9%, accounting for 50% of the Hispanic population), whereas it is largely uninfluenced by the high prevalence among mothers who identified as Cuban (90.9%, accounting for 3% of the overall Hispanic population). Disaggregation allows for a more detailed description of subgroups of smaller size.

As in the findings in our case study of breastfeeding, previous studies of other health behaviors, conditions, and outcomes also suggest obscured disparities caused by aggregated analysis. In a study of racial disparities in COVID-19, researchers disaggregated case rates among the Native Hawaiian or Other Pacific Islander racial group twice: first, researchers disaggregated Native Hawaiian people and Pacific Islander people, and then they further disaggregated Pacific Islander data to 7 more specific subgroups (12). When aggregated, COVID-19 case rates among Native Hawaiian or Other Pacific Islander people were higher than all other OMB racial groups (White, Black, American Indian or Alaskan Native, and Asian). After disaggregation, it became apparent that COVID-19 case rates among Native Hawaiian or Other Pacific Islander people were driven by high case rates among only 2 Pacific Islander subgroups (which were of moderate population size). The authors concluded that aggregated analysis obscured disparities identified among certain subpopulations.

It is well established that heterogeneity exists within the broad racial and ethnic categories outlined by OMB and typically used in public health research. Within each broad category is a range of languages, nationalities, immigration and refugee histories, nativity statuses, levels of acculturation, and socioeconomic backgrounds. If race and ethnicity data are only collected or reported in aggregated categories, the level of granularity needed to make decisions about the varied health needs of different racial and ethnic groups may be lacking (24). When detailed data do not exist, this invisibility and lack of representation for certain populations is known as data inequity, which is seen as a critical source of health inequity (25). For public health researchers, birth certificate data are a timely and robust source of maternal and infant health data that allow for detailed examination of race and ethnicity.

Although data disaggregation is an important component of improving health equity, barriers exist to collecting and disaggregating data. A 2021 study reviewed disaggregated data collection methods in large, US population–based health surveys for Asian American and Native Hawaiian or Other Pacific Islander groups (18). Barriers cited included the lack of interest on the part of the survey administrator, inadequate funding, lack of space on questionnaires, as well as methodologic challenges, such as the inability to obtain an adequate sample size and poor questionnaire design. The study’s authors found that although large national surveys and vital statistics may include Asian American and Native Hawaiian or Other Pacific Islander subpopulations in data collection, sample sizes are sometimes too small to permit meaningful data analysis. There is also the risk of disclosure with small sample sizes. The authors concluded that survey administration efforts to improve disaggregated health data are challenging, but when completed, have led to a proliferation of new knowledge on the health of subgroups.

Although we have attempted to disaggregate breastfeeding initiation by racial and ethnic groups, we were unable to disaggregate some heterogeneous groups further by using publicly available data (though this would be possible to examine in NCHS’s Research Data Center). For example, the lowest prevalence of breastfeeding initiation among the aggregated Asian and Native Hawaiian or Other Pacific Islander racial groups was in the subgroups of “Other Asian” and “Other Pacific Islander,” respectively. The “Other Asian” category is heterogeneous, and includes mothers having origins in Bhutan, Cambodia, Malaysia, Nepal, Pakistan, Thailand, and elsewhere, in addition to mothers reporting 2 or more Asian groups (eg, Japanese and Korean) (15,18). Similarly, the “Other Pacific Islander” category is diverse and composed of mothers identifying as Chuukese, Fijian, Marshallese, Palauan, Polynesian, Tahitian, Tongan, and more (15). This issue of heterogeneous subgroups is also present in the Hispanic ethnicity variable, where the “Central or South American” group could span mothers with origins across thousands of miles and distinct cultures. Although we can disaggregate breastfeeding initiation rates by certain Asian, Native Hawaiian or Other Pacific Islander, and Hispanic subgroups, additional disaggregation may be warranted.

Racial and ethnic groupings are based on ever-changing cultural norms; therefore, data collection of race and ethnicity is constantly evolving. For example, in early 2023, major changes were proposed to update and revise the OMB race and ethnicity standards to combine the race and ethnicity questions and add Middle Eastern or North African as a category (26). Within each larger race or ethnicity group, there is the opportunity to give further detail; for instance, within the Middle Eastern or North African group, additional selection(s) can be made for Lebanese, Iranian, Egyptian, Syrian, Moroccan, or Israeli, or the respondent can write in a response. These proposed changes demonstrate the evolution of race and ethnicity data collection and opportunities to improve it.

Racial and ethnic disparities in breastfeeding have been documented previously (5–7), and breastfeeding initiation rates by more granular racial and ethnic groups in 2 states (California and Hawaii) showed similar patterns to this analysis of data from 48 states and the District of Columbia (27,28). In our analysis, to produce prevalence estimates that adjust for maternal and infant characteristics, which can affect breastfeeding initiation and are distributed differently among racial and ethnic subgroups, we see disparities within Asian American, Native Hawaiian or Other Pacific Islander, and Hispanic populations. Previous research has described common barriers to breastfeeding, which may vary by racial or ethnic group. These barriers might include social norms, employment and childcare challenges, barriers related to health services, poor family and social support, embarrassment, lactation problems, and lack of knowledge (29).

### Strengths and limitations

Strengths of this study include its use of data on all births from 48 states and the District of Columbia. Furthermore, maternal race and maternal ethnicity data from the birth certificate are more detailed relative to all other breastfeeding data sources. The findings reported here are subject to at least 5 limitations. First, birth certificates do not include information on exclusivity and duration of breastfeeding, which are important indicators of optimal infant nutrition. Second, breastfeeding initiation data might be misclassified. Although a comparison of birth certificates to medical records across 8 hospitals in 2 states found high sensitivity of breastfeeding initiation, moderate false discovery rates suggest that discrepancies might exist between medical records and birth certificates (30). However, overall rates are generally consistent with other national data sources. Third, birth certificate data reliability and validity are not known to have been assessed across racial or ethnic groups. Misclassification of breastfeeding data might vary by race or ethnicity. Fourth, estimates are not nationally representative because births from California and Michigan (representing ~15% of US births) cannot be included in analyses. Lastly, we were unable to disaggregate the largest racial categories by population size (eg, White, Black) by using publicly available data because the current version of the birth certificate does not include subgroups for these racial groups. Therefore, we are limited in our understanding of potential variations in breastfeeding initiation among these racial groups.

### Conclusions

Data disaggregation by detailed racial and ethnic subgroups paints a more detailed picture of health (25), as we have demonstrated by using data on breastfeeding initiation. Disaggregation can ensure that populations that have been historically masked in public health surveillance and research are more visible (8), allowing for health and social services approaches to address specific needs and create solutions to eliminate health disparities in outcomes, such as breastfeeding initiation.
